# Bronchiolar adenoma with diffuse pulmonary nodules: a extremely rare case report and review of literature

**DOI:** 10.1186/s12890-020-01228-1

**Published:** 2020-07-14

**Authors:** Yajing Sun, Min Liu, Zhongmin Jiang, Baojiang Li

**Affiliations:** Department of Pathology, Tianjin Fifth Central Hospital, 41 Zhejiang Road, Tanggu District, Tianjin, 300450 China

**Keywords:** Bronchial adenoma(BA), Diffuse pulmonary nodules, Pathological features, Immunophenotype, Differential diagnosis, Case report

## Abstract

**Background:**

Bronchiolar adenoma(BA) is a recently recognized, rare tumor of the bronchioles. It can be divided into proximal and distal types according to the proportion of mucinous and ciliated cells on the luminal surface. BA is often misdiagnosed because it has similar ultrasonographic, gross and histological presentations as other diseases. Here, we report a rare case of BA characterized by many fused nodules.

**Case presentation:**

A 68-year-old woman attended the Tianjin Taida Hospital surgical Clinic mainly because of “intermittent cough for >1 month”. Chest computed tomography (CT) showed multiple solid nodules in the upper and lower left lung. The nodules had irregular outlines, with a maximum diameter of 65 mm. A double needle lung biopsy specimen was removed guided by ultrasound under local anesthesia. Histologically, the biopsy specimen was finally diagnosed as the distal type of BA.

**Conclusion:**

BA with diffuse pulmonary nodules is rare. Diagnosis of BA needs comprehensive analysis of imaging, gross specimen analysis, histopathology, and immunohistochemical staining to make a correct diagnosis and avoid misdiagnosis. There are few studies on prognosis, which needs close follow-up and more data accumulation.

## Background

Bronchiolar adenoma(BA) is a recently recognized, rare tumor of the bronchioles [1], and most pathologists and clinicians are not aware of it. BA is often misdiagnosed because it has similar ultrasonographic, gross and histological presentationsas other diseases. Therefore, improving the diagnostic accuracy of BA is important for the follow-up treatment and prognosis.

Previous reports state that BA is a rare, benign entity that is characterized by a double-layer cell structure with specific immunophenotype. It can be divided into proximal and distal types according to the proportion of mucinous and ciliated cells on the luminal surface. The main body of the tumor (usually single) is located in the lung parenchyma outside the bronchioles, BA has a clear boundary and no capsule, with papillary or flattened growth along the alveolar wall. Recently, it has been proposed that BA is a tumor with undetermined malignant potential [2, 3]. Here, we report a rare case of BA characterized by many fused nodules, and review the relevant literature.

## Case presentation

On March 11, 2020, a 68-year-old woman attended the Tianjin Taida Hospital surgical Clinic mainly because of intermittent cough for > 1 month. She had a 2-year history of hypertension controlled by medication, and no history of smoking or alcohol consumption. She had an intermittent cough with white sticky sputum, without any other symptoms or signs. On the same day, the patient was referred to the Fifth Central Hospital of Tianjin. Here vital signs were: body temperature 36.2 °C, pulse 86 beats/min, respiration rate 18 breaths/min, blood pressure 142/85 mmHg, and she was fully conscious. There was no visible enlargement of her superficial lymph nodes. She had no cyanosis, percussion pain in the sternum, or abnormal respiratory movements. She had coarse breath sounds in both lungs, no pleural friction sounds, and audible inspiratory crackles in the left lung. Heart sounds were strong and regular, with no pathological murmurs in any valve auscultation area. Carcinoembryonic antigen (CEA) was 8 ng/ml (normal range 0–5 ng/ml) and non-small cell lung cancer associated antigenCYF21–1 was 13.31 ng/ml (normal range 0.1–3.3 ng/ml). Other biochemical indicators were within the normal range.

**Fig. 1:**
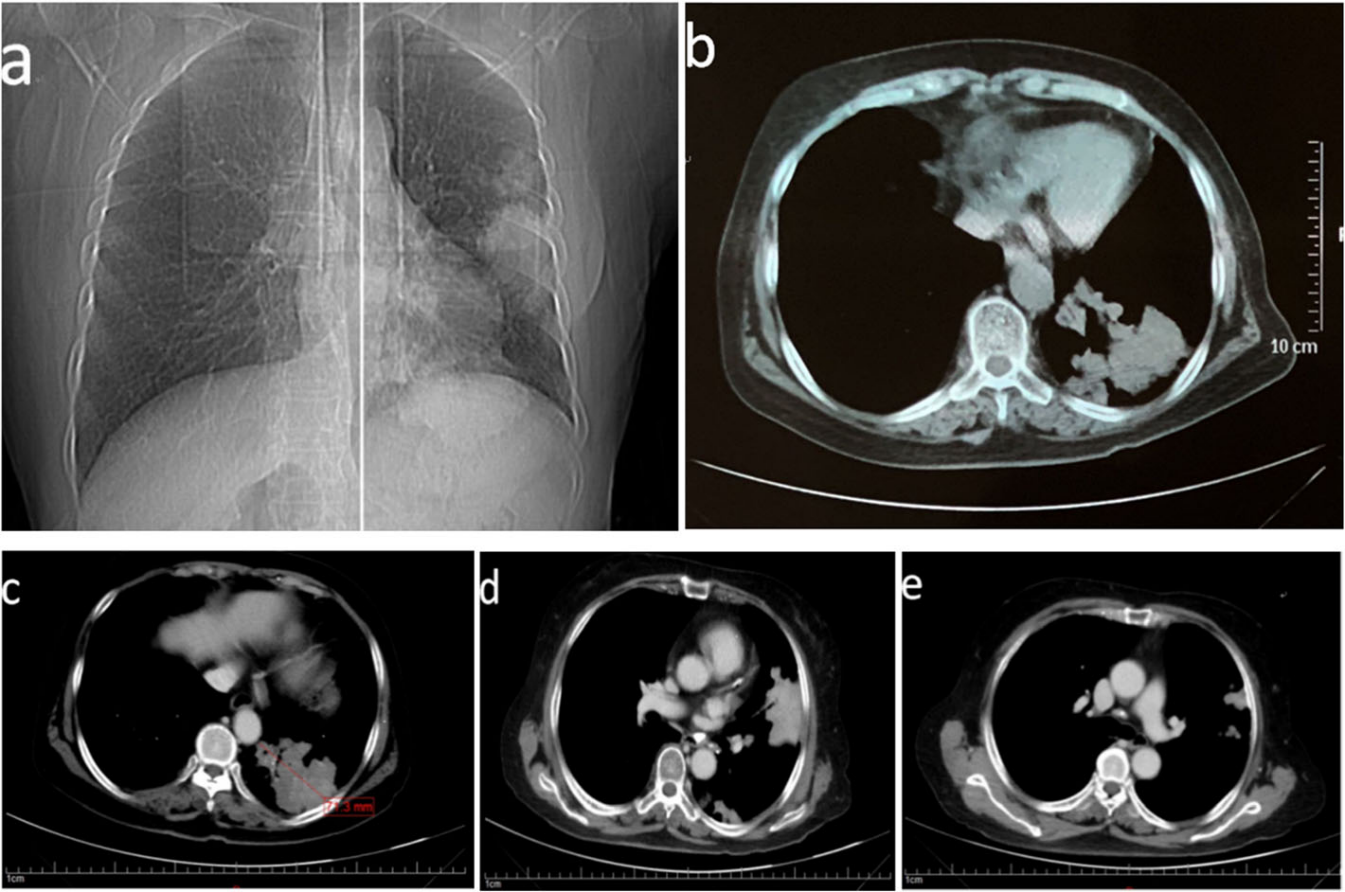
Imaging examinations: **a** Chest radiography:nodules in the upper and lower segments of the left lung. **b** CT: “cauliflower-like tumor” formed by fusion of multiple nodules. **c** CT: soft tissue mass with diameter of 71.3 mm. **d** CT: multinodular confluent tumor close to the pleura. **e** Some small nodules in the lungs

The patient underwent a series of imaging examinations (Fig. 1). Chest radiography on March 11, 2020 showed several nodules in the upper and lower segments of the left lung (Fig. 1a). Chest computed tomography (CT) on March 12, 2020 showed multiple solid nodules in the upper and lower left lung (Fig. 1b). The nodules had irregular outlines, with a maximum diameter of 65 mm, and some had a central cavity. A diagnosis of lymphoma was considered. Enhanced chest CT on March 16, 2020 (Fig. 1c–e) showed multiple “cauliflower like”, soft tissue masses in the left lung, partially fused, with a maximum diameter of 71.3 mm and a CT value of ~ 36 HU. Enhanced CT showed enhancement with a maximum value of 60 Hu. The boundary between the mass and pleura was not clear, and the mass could not be separated from the pleura. There was irregular thickening of the pleura and no enlargement of lymph nodes. A malignant tumor could not be excluded.

To clarify the pathological diagnosis, the patient underwent ultrasound-guided percutaneous needle biopsy of the left lung under local anesthesia on March 16, 2020. After consultation with our own and other hospitals(diagnostics were performed by Tianjin Fifth Central Hospital, Tianjin Cancer Hospital and Peking Union Medical College Hospital), the biopsy specimen was finally diagnosed as BA according to histomorphology and immunophenotype.

Wedge resection with safe margins may be the best treatment option for BA patients. However, if the lung is full of diffuse nodules, the whole lung may need to be removed, which seriously affects quality of life. Our patient was treated conservatively and followed up regularly. She was discharged on March 28, 2020.Her symptoms were managed through regular normal activities of daily living, strengthening respiratory protection, avoiding respiratory infection, and drug treatment to relieve dyspnea and cough.

**Fig. 2:**
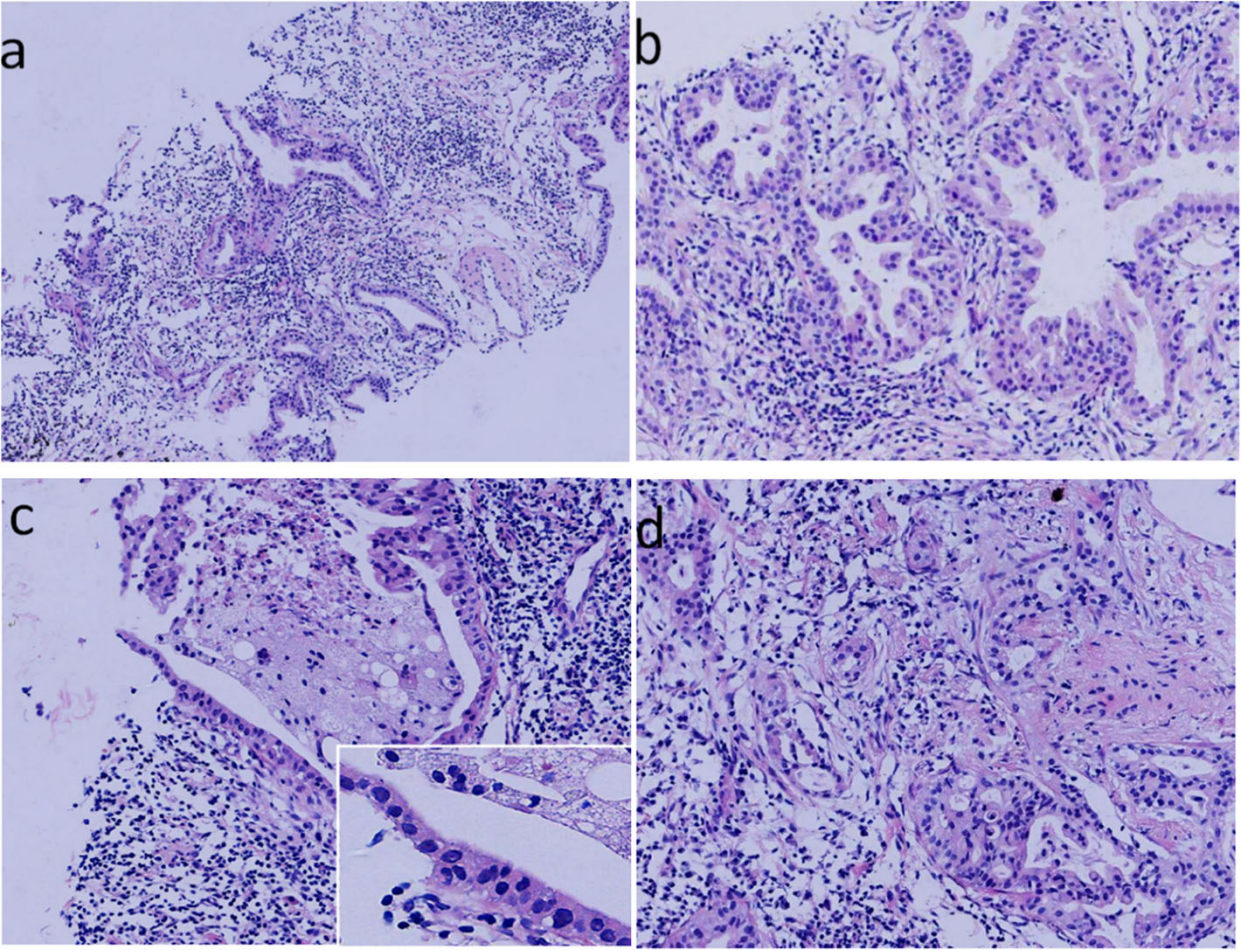
Hematoxylin-and-eosin-stained sections of lung masses. **a** Irregular glands grew in a significant inflammatory background. **b** Micropapillae were occasionally formed by nonciliated cells (such as type II alveolar cells and club cells). **c** Ciliated columnar cells were identified occasionally. **d** Irregular glands, similar to adenocarcinoma, with a double cellular structure

To clarify the diagnosis, a double needle lung biopsy specimen was removed, 15 mm long, 1 mm in diameter. It was solid and gray white. Microscopically, there were irregular adenoid structures in a background of fibrosis and inflammation (Fig. 2a). The lesion had a two-layer cellular structure consisting of a basal and luminal layer, and had a lack of papillary structures. The micropapillae occasionally formed by non-ciliated cells germinated and burrowed into the alveolar cavity(Fig. 2b). The luminal layer was composed of type II alveolar cells and club cells. However, ciliated columnar cells and mucous cells in some flat areas could still be identified (Fig. 2c).The appearance was similar to adenocarcinoma in some areas, with alveolar destruction and stromal widening. Through careful observation, we found that the main reason for the stromal widening was edema and inflammatory infiltration. Unlike adenocarcinoma, there was a lack of thick collagen fibers (Fig. 2d). Tumor cells lacked atypia, mitosis and necrosis. A layer of material resembling the basement membrane surrounded the basal layer. The most important feature was a continuous layer of basal cells around the luminal layer.

**Fig. 3:**
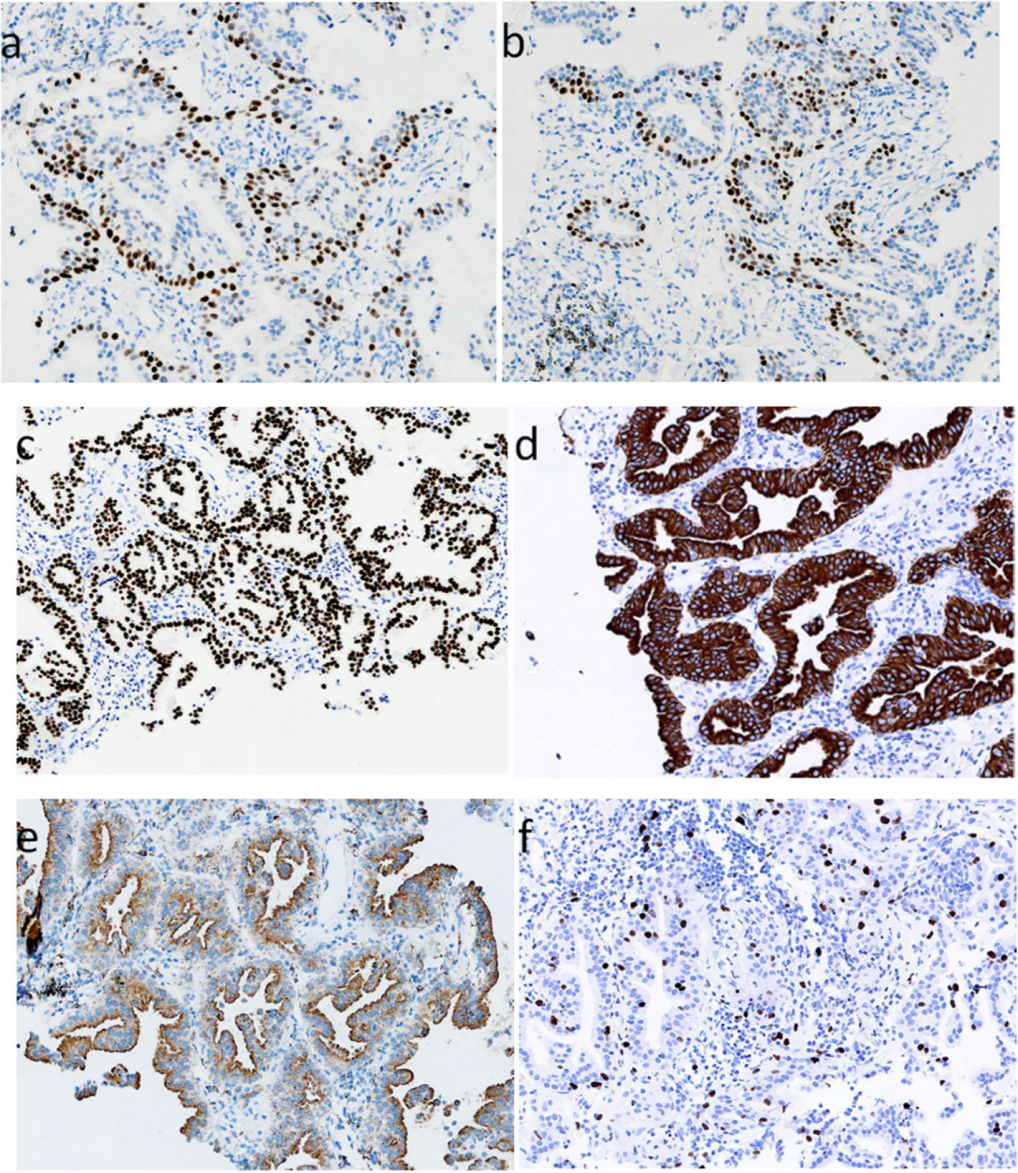
Immunohistochemical staining of lung masses. **a** Positive expression of p63 in basal cells. **b** Positive expression of p40 in basal cells. **c** Positive expression of TTF-1 in luminal cells and some basal cells. **d** Positive expression of CK7 in luminal cells. **e** Positive expression of Napsin A in luminal cells. **f** The tumor showed a < 10% Ki-67 proliferation index

Immunohistochemical staining for p63, p40 and cytokeratin (CK)5/6 clearly showed the continuity of the basal cells (Fig. 3a, b). The luminal cells and some basal cells stained positive for thyroid transcription factor (TTF)-1. The luminal cells expressed immunological markers of the terminal bronchioles (CKpan, CK7, Napsin A and CEA). A small number of mucous cells stained positive for periodic acid Schiff. The tumor showed a < 10% Ki-67 proliferation index (Fig. 3). Finally, the case was pathologically diagnosed as BA of distal type.

## Discussion and conclusion

BA was first reported in 2017 by Chang and colleagues [1]. BA can be divided into proximal and distal types, with most cases being proximal type. Both types present with a bilayer architecture composed of luminal cells and continuous basal cells [2, 3]. The proximal type is also known as ciliated muconodular papillary tumor, and its luminal cells are composed of ciliated columnar cells and mucinous cells [4]. The luminal cells of the distal type are composed of type II alveolar cells and club cells. The immunophenotypes of the two types of BA are similar, with some differences. The luminal cells of the proximal type are negative or weakly positive for TTF-1 and Napsin A, but the distal type is strongly positive. Basal cells of both types are positive for p40, p63 and CK5/6, and weakly positive for TTF-1 [5, 6]. The neoplastic nature of BA is shown by genetic alterations such as BRAF V600E, EGFR, ALK and KRAS. However, these alterations are not specific for BA [7–10].

Previously, BA was often misdiagnosed. Differential diagnosis between benign and malignant conditions is important because of their different treatment and outcomes. Frozen section diagnosis is especially challenging.

Invasive adenocarcinoma and BA are easily misdiagnosed because of their irregular adenoid structures and widened stroma. BA contains basal cells and ciliated cells, while atypia and pathological mitosis are absent. Invasive adenocarcinoma has no basal or ciliated cells, and atypia and pathological mitosis are always present. Although both tumors have widened stroma, inflammation and edema can be found in BA, while more collagen fibers can be found in invasive adenocarcinoma. p40, p63 and CK5/6 are positive in BA but negative in invasive adenocarcinoma. Expression of Ki-67 is low in BA but high in invasive adenocarcinoma.

It is difficult to distinguish bronchiolar metaplasia and BA microscopically because they have similar morphology and immunophenotype. Patients with bronchiolar metaplasia often have other background diseases, such as interstitial lung disease or organic pneumonia. BA forms a mass that is visible in imaging, whereas bronchiolar metaplasia usually has a punctate focus and does not form a mass that is visible in imaging. Therefore, imaging examination is important for the differential diagnosis of these two diseases.

Mixed squamous cell and glandular papilloma is a benign tumor with basal, ciliated, squamous and mucinous cells, and it also sometimes produces mucus. The tumor is located in the bronchial lumen, which is different from BA. The papillary structure can be found easily and the axis is rich in fiber and blood vessels.

Sclerosing pneumocytoma is a benign tumor with two types of cells: surface epithelial cells and stromal round cells. It has at least three of the following four structures: papillary, solid, hemangioma-like, and sclerotic structures. Sclerosing pneumocytoma cells often have some atypia, while BA has no atypia. In sclerosing pneumocytoma, surface epithelial cells are positive for CK7, glandular epithelial marker CAM5.2 and Napsin A, and stromal round cells are positive for vimentin. Surface epithelial cells and stromal round cells are positive for epithelial membrane antigen and TTF-1.

Mucoepidermoid carcinoma is composed of many types of cells, including epidermoid, intermediate and mucinous cells. When the glands of BA are sieve-like with mucus secretion, they need to be differentiated from mucoepidermoid carcinoma. Mucoepidermoid carcinoma is malignant and invasive and the boundary is not usually clear, which is different from BA. Immunohistochemistry shows that CK7, p63 and p40 are positive in mucoepidermoid carcinoma, but TTF-1 is negative.

Previous studies have shown that BA is a rare, benign tumor. However, some recent studies have shown that BA has undetermined malignant potential and can recur and metastasize. Therefore, it is important to improve the recognition and diagnostic accuracy of BA for follow-up treatment and prognosis. Diagnosis of BA needs comprehensive analysis of imaging, gross specimen analysis, histopathology, and immunohistochemical staining to make a correct diagnosis and avoid misdiagnosis. Our case had diffuse pathological changes in the lungs, which could easily have been misdiagnosed as a malignant tumor. Correct diagnosis avoids unnecessary surgery, radiotherapy and chemotherapy. We followed up our patient until June 6, 2020. Western medicine only partially relieved the symptoms of dyspnea and cough. Therefore, the patient tried traditional Chinese medicine. In the morning, there was a little white phlegm, with only occasional or almost no yellow phlegm. Chest CT on May 16, 2020 was used to monitor lung changes, but there was no significant change compared with preoperative findings.

BA with diffuse pulmonary nodules is rare. There are few studies on prognosis, which needs close follow-up and more data accumulation.

## Data Availability

The data used and/or analyzed during the current study are available from the corresponding author on reasonable request.

## Abbreviations

BA: Bronchiolar adenoma
CT: Computed tomography
PBM: Bronchiolar metaplasia
CMPT: Ciliated muconodular papillary tumors
PMSGP: Mixed squamous cell andglandular papilloma
CEA: Carcinoembryonic antigen
TTF: Thyroid transcription factor
CK: Cytokeratin
